# Rheumatoid arthritis patient perceptions on the value of predictive testing for treatments: a qualitative study

**DOI:** 10.1186/s12891-016-1319-x

**Published:** 2016-11-08

**Authors:** Kanta Kumar, Sarah Peters, Anne Barton, on behalf of MATURA

**Affiliations:** 1 School of Health Sciences, University of Manchester, Manchester, M13 9PL UK; 2 Arthritis Research UK Centre for Genetics and Genomics Centre for Musculoskeletal Research, Institution of Inflammation and Repair, University of Manchester, Manchester, M13 9PL UK; 3 Manchester Centre for Health Psychology, School of Health Sciences, Manchester, M13 9PL UK; 4 NIHR Manchester Musculoskeletal BRU, Central Manchester Foundation Trust, Manchester Academic Health Sciences Centre, Manchester, UK

**Keywords:** Rheumatoid arthritis, Predictive testing, Patient perspective

## Abstract

**Background:**

Rheumatoid arthritis (RA) is a long term condition that requires early treatment to control symptoms and improve long-term outcomes. Lack of response to RA treatments is not only a waste of healthcare resources, but also causes disability and distress to patients. Identifying biomarkers predictive of treatment response offers an opportunity to improve clinical decisions about which treatment to recommend in patients and could ultimately lead to better patient outcomes. The aim of this study was to explore the understanding of and factors affecting Rheumatoid Arthritis (RA) patients’ decisions around predictive treatment testing.

**Methods:**

A qualitative study was conducted with a purposive sample of 16 patients with RA from three major UK cities. Four focus groups explored patient perceptions of the use of biomarker tests to predict response to treatments. Interviews were audio-recorded, transcribed verbatim and analysed using thematic analysis by three researchers.

**Results:**

Data were organised within three interlinking themes: [1] Perceptions of predictive tests and patient preference of tests; [2] Utility of the test to manage expectations; [3] The influence of the disease duration on take up of predictive testing. During consultations for predictive testing, patients felt they would need, first, careful explanations detailing the consequences of untreated RA and delayed treatment response and, second, support to balance the risks of tests, which might be invasive and/or only moderately accurate, with the potential benefits of better management of symptoms.

**Conclusions:**

This study provides important insights into predictive testing. Besides supporting clinical decision making, the development of predictive testing in RA is largely supported by patients. Developing strategies which communicate risk information about predictive testing effectively while reducing the psychological burden associated with this information will be essential to maximise uptake.

## Background

Rheumatoid arthritis (RA) is a long term condition that requires early treatment to control symptoms and improve long-term outcomes [1]. Lack of response to RA treatments is not only a waste of healthcare resources [2], but also causes disability and distress to patients. Treating RA is not a uniform approach and significant non-response rates are reported for all the commonly used medications including both non-biologic and biologic disease modifying anti-rheumatic drugs (DMARDs). It would be ideal to target available treatments to individual patients based on their likelihood of response to ensure that remission is achieved in the fastest time. However, currently, effective treatments can only be identified through trial and error, often over prolonged periods. If it was possible to predict with a reasonably high degree of certainty that a treatment for RA is likely to be effective for an individual patient, treatment response rates and time to remission may improve whilst costs due to wastage of healthcare resources would be reduced.

Identifying biomarkers predictive of treatment response offers an opportunity to improve clinical decisions about which treatment to recommend in patients and could ultimately lead to better patient outcomes [3]. Currently, methotrexate is typically prescribed as the first choice DMARD to treat RA either as mono or combined therapy but by two years, up to 45 % of patients have moved to other therapies [4]; patients who fail to respond to methotrexate can be prescribed another DMARD, or a biologic drug, which modulates the immune system and/or inflammatory pathways to alleviate disease. However, up to 25 % of patients prescribed anti-TNF and 40 % of patients given rituximab biologic therapies show inadequate response by 6 months of treatment [5, 6]. This is important because early, effective intervention has been consistently shown to improve long-term outcomes including disability and joint damage. Therefore, there appears to be an unmet clinical need to identify biomarkers that would allow clinicians to select the treatment most likely to work for a patient, before the treatment is administered, based on some form of test; such a test may incorporate a number of factors including demographic, and clinical measures but may also use results from blood and/or tissue sample tests. Identified factors are called predictive biomarkers or theranostic biomarkers and are used extensively in the cancer therapeutic field; for example, expression of the estrogen receptor (ER) gene predicts response to tamoxifen in patients with breast cancer and such tests are used routinely to help select the most appropriate treatments for individual patients [6]. In RA, no predictive tests are yet available but numerous research studies are underway with the aim of identifying such biomarkers. The cost of testing will vary according to the particular marker identified but a recent study investigating the costs of a blood based testing of drug levels to anti-TNF biologic drugs estimated that it would cost £152.52/patient (range: £147.68 to 159.24) [7]. However, before making recommendations about introducing such theranostic testing into the clinical setting, it is important to understand the patient perspectives and preferences. This requires an understanding of the range of effects that predictive testing can have on RA patients. This study explored the factors that might influence a patients’ decision to agree to different tests.

## Methods

A qualitative study was undertaken in which explorative focus groups were conducted with RA patients from Manchester, Birmingham and London, UK. Patients with a diagnosis of RA and who were taking synthetic or biologic DMARDs were recruited from the National Rheumatoid Arthritis Society (www.nras.org.uk) and through established patient user groups. Patients were selected purposively to ensure a mixture of age, gender and employment status. Invitation letters were sent to suitable patients and those that agreed to take part in the study were invited to a focus group interview. All study participants provided their written consent.

Four focus groups were conducted with 16 patients at three sites (The University of Manchester, the University of Birmingham and the Centre for Experimental Medicine & Rheumatology, London) in areas designated for research. By working with the MATURA patient advisory group (www.matura.whri.qmul.ac.uk), we developed a topic guide for the interviews where two scenarios were presented to patients: [1] use of synovial biopsy-based testing and [2] use of blood tests to inform treatment selection each with different levels of accuracy for predicting future response. Patients were briefly reminded of the background and aim of the study, were asked to reflect on the scenarios and their views on decision making around the tests explored. The interviewer used open questions to encourage emergence of participants’ ideas.

All focus groups interviews were audio-recorded, transcribed verbatim, anonymised and analysed using thematic analysis where saturation of themes were determined [8]. The data gathered from the focus groups were analysed by thematic analysis [9] in which initial analytical summaries of the focus group interviews were coded line by line; the coding framework was discussed by three researchers and coding categories that lacked concordance were absorbed back into the coding framework. The initial codes were then grouped into the most frequently occurring categories. The core themes extracted and presented here focus on perceptions of predictive testing and patients’ views about the understanding of these tests. Data collection and analysis occurred in parallel to allow for an assessment of when thematic saturation had been achieved, i.e., the point at which no new ideas emerged [8].

## Results

In the four focus groups, (Table 1). The majority (*n* = 13) of the participants were female. The age range of the participants was 26–80 years old and disease duration was 3–34 years. Eight participants had retired, four were semi-retired and four were in full-time employment.

**Table 1:** Details of RA patients who took part in the interviews

Gender 13 female, 3 male
Age range and mean Range: 26–80 mean: 42
Employment status employed full time: 5, self-employed; 1, retired; 4 retired due to RA;6
Range of year of disease Range: 3–34 years
Interview Place Birmingham; 3, London; 5, Manchester; 8 participants

The data are organised into three superordinate themes: [1] Perceptions of predictive tests and patient preference of tests; [2] Utility of the test to manage expectations; [3] The influence of the disease duration on take up of predictive testing. Each theme is illustrated with quotations extracted from the transcripts.

### [1] Perceptions of predictive tests and patient preference of tests

Participants’ perceptions towards predictive testing were largely positive as participants viewed predictive testing as potentially helpful to impart new information about which treatment could best control their symptoms, improve quality of life and reduce disability. Participants were strongly motivated to reduce delays in finding an effective treatment, reduce the likelihood of side effects and achieve remission more quickly.

Participants expressed concerns about the pain related to a synovial biopsy procedure but balanced this anxiety with the benefit of assumed increased predictive accuracy. Hence they were prepared to undergo a biopsy if this increased the accuracy of the test. Indeed the benefits of the possibility of better management of their condition were so valued that this trade-off was acceptable even when the probability of finding an accurate treatment was low (Table 2 Quotations 1–9).

**Table 2:** Patient Quotes

Themes Perceptions of predictive tests and patient preference of tests	Quote 1: *I wouldn’t hesitate if there was any option to do anything at all that would be likely to make your life better. *[female, left work due to RA] Quote 2: *I would have loved to have continued my job, and had I had this kind of test done I would probably still be in my job*. [female, retired] Quote 3. *Because there’s quite a lot of waiting around, even after you’re prescribed a specific drug, and then even more because I’m just going through the process of swapping them again …So anything that reduces that amount of time that you have to wait and knowing that when you are on the drug it’s more likely to work rather than trial and error and just seeing whether it does work would definitely be something that I would consent to.* [female, full-time employment] Quote 4: *My answer to that would be yes the biopsy would be more painful to go through but, if that was the best your test had produced in the biopsy…say 50 % or 60 % then I would go with that in the hope that you would refine and improve the quality of life.* [female, retired] Quote 5: *I think the biopsy would give you a better indication, rather than a blood test.* [male retired] Quote 6: *I would still take the drug if the test indicated that it only had a 20 % chance of effectiveness.* [male, full-time employment] Quote 7: *“it will mean that you don’t have to go through years and years of trying different drugs, I think then I wouldn't even question the biopsy test”*. [female, retired] Quote 8: *“I think, for me, the big thing would be accuracy and how accurate it is; if the biopsy tells you more then that’s what I should go for.* [female, full-employment] Quote 9: *Yeah, similar. I mean, I guess, it depends in part, how accurate you think it could be… Like you say, it’s not such a major procedure [referring to biopsy] or whatever, but would be worth having a go at it”.* [female, full-employment]
Utility of the test to manage expectations	Quote 10: *I think it’s probably more of a personality sort of thing. I would imagine two people hear the same information and they react differently* [male retired] Quote 11: *I’m a research scientist, so for me, I’m just nosy. The more I know about what’s going on, the more I can manage my expectations. But I guess that’s not true for most patients. So it’s trying to think of what other people would say*…[female, full-employment] Quote 12: *I can see if you’ve pinned your hopes on something and it comes back ten per cent, this isn’t going to work for you, then yes, you might want to speak to someone. Yeah, because you’re sort of…so you almost go through a grief process, don’t you?* [female, full-employment]
The stages of illness on decision making for predictive testing	Quote 13: *I think it goes without saying. I think a test such as that, if you’re new to it you’re not aware of the journey that you’re going to undertake with rheumatoid.* [male, retired] Quote 14: “*it’s barely an operation, and it’s going to help my clinicians have a better understanding of my profile, absolutely, 100 %. But it’s a question of understanding the potential journey. If you don’t understand the impact of life then you think, well, why is it necessary?”.* [female left work due to RA] Quote 15: *“I think anything…as a newly diagnosed patient, or going through that diagnosis process, any sort of conversation about medications is helpful and if tests are the way forward to predict and control disease then that should be explained”* [female full-employment] Quote 16: “*Right at the beginning you don’t know what it’s going to be like, what the side effects of the medication are going to be like, so as long as that is all explained to the patients this is what happens with rheumatoid arthritis then the new ones will have a better idea*”. [female, left work due to RA] Quote 17: “*The group of us here are clearly enlightened and we understand what’s involved. Some are educated, some are partially educated and some are highly educated, but, from my experience, the majority have little understanding of what their condition is*”. [male, retired] Quote 18: “*I mean, it might be probably better if you get new patients to have them all in one clinic where somebody could explain what the condition is that they have once they’ve had their blood tests, and then seen individually by a consultant following that group thing where they’ve been told what they’ve got, what treatments there are, what further tests they need*” [female, left work due to RA]

### [2] Utility of the test to manage expectations

Participants expressed the view that there may be inter-individual variation in the interpretation of test results. It was argued that people may react differently towards the results of tests even though they receive the same test result and those who place a high expectation on the test result may be more disappointed and need more reassurance if the test result showed a low probability of efficacy of a particular drug. Some participants thought that knowing that the test might have a low accuracy may help them to manage their expectations. However, if an accurate test produced a result indicating a low likelihood of benefit for a particular treatment, that could create a sense of loss about how and to what level the symptoms might be controlled. Regardless, participants felt that they would need additional support to understand the risk and cope with the emotional impact of a test that predicted a low probability of response (Table 2 Quotations 10–12).

### [3] The influence of the disease duration on take up of predictive testing

Amongst participants, there was a view that the stage of the disease course could influence a patient’s willingness to engage in predictive testing. Participants were concerned that the value of a predictive theranostic biomarker test may not be recognised by those with recently diagnosed RA; by contrast, those with established RA might value the predictive testing more in determining their treatment plan since they would have had experience of the fluctuant condition and the extent to which it impacts on life. It was thought that those with limited RA experience would require more convincing information and careful explanations about the utility of predictive testing and how that would add value in determining a timely treatment plan (Table 2 Quotations 13–18).

## Discussion

This is the first study to explore the understanding of and factors affecting RA patients’ decisions around tests that predict likely treatment efficacy. Whilst such tests are largely still hypothetical, there are a number of initiatives to identify accurate and reliable theranostic biomarkers and it is reassuring to note, therefore, this study evaluated patient perceptions towards the concept of predictive testing, not the tests themselves, these perceptions were very positively overall. Even though synovial biopsy was viewed as being a painful and invasive procedure, and some had concerns about tests with only low-moderate accuracy, these were outweighed by the potential benefits of accessing new information that could reduce delays in finding an effective treatment. One recommendation that emerged was the need for information provision before predictive testing, particularly about the consequences of uncontrolled disease activity so that the risks of the test could be balanced with the current trial-and-error approach of therapy selection.

In keeping with previous studies investigating the risk of developing inflammatory diseases [10], negative views were expressed if the test failed to meet the patients’ expectations. Thus, some concerns were raised about the effect of learning that a test predicted a low probability that a treatment would be effective as this may cause anxiety and be viewed as a negative. Moreover, this could be particularly distressing for patients with newly diagnosed disease. By contrast, a test result showing a high probability of efficacy may improve adherence levels. These findings highlight the need for careful support to minimise distress for patients and map onto similar issues that have been explored in studies investigating the views of genetic testing in people at risk of inflammatory disease [11–14]. In those studies, a staged approach has been recommended [10, 15] incorporating provision of information prior to testing, providing help to interpret the results [13, 16] and finally offering psychological support [17]. In general, patient understanding of predictive testing is likely to be variable [15] and we recommend that clinics considering offering patients predictive testing pay particular attention to the impact that the negative result might have on the individual patient. Patients at different stages of their disease might react differently to predicative testing. For example, patients with established disease duration might be more willing to try tests since they would have had more experience with treatments that might not have worked effectively. Future work should be conducted in newly-diagnosed patients, who were not well-represented in the current study, to determine whether the factors influencing on the decision to undergo predictive testing are the same or not.

This study has a number of limitations. Firstly, our access to patients was via patient user groups and charity organisations. These patients might be very proactive in their views and be motivated to support research and innovative approaches. The findings presented may not, therefore, fully reflect the range of views related to predictive testing in the general clinic setting. Given the finding that stage of illness may be a factor in acceptability of predictive testing, exploring this further in those newly diagnosed would be an important next step. A second limitation was that the majority of patients were female Caucasian. Previous work in rheumatology has been criticised for underrepresenting the male perspective [18] and those of Black and minority ethnic groups. Whilst efforts were made to engage these more difficult to reach groups, the focus groups are unlikely to be fully representative. A full understanding of the cultural barriers (including ethnicity, gender and religion) related to predictive testing was not captured and is an area for further research as the support required in these populations may differ. Despite these limitations our study also has a number of strengths: first, patients were sampled from three different centres across the UK; second, it is the first that has attempted to explore the views of patients about predictive testing for RA treatments; third, we have captured recommendations made by patients that can be implemented to alleviate patient concerns if/when predictive testing reaches the clinical setting and finally, we have demonstrated the importance of individual circumstances and the clinical encounter in the appraisal of predictive testing for treatment response in RA clinics.

## Conclusions

Predictive testing to better inform treatment selection decisions is welcomed by RA patients, but support is needed during the decision making process to help manage the impact of a result suggesting a low probability of treatment efficacy, particularly for patients early in the illness trajectory. Developing strategies which healthcare professionals can use to communicate risk information effectively while reducing the psychological burden associated with this information is essential.

## Abbreviations

DMARDs: Disease modifying anti-rheumatic drugs
RA: Rheumatoid arthritis
